# 8-Iodo­quinolinium chloride dihydrate

**DOI:** 10.1107/S1600536808031528

**Published:** 2008-10-04

**Authors:** Jung-Ho Son, James D. Hoefelmeyer

**Affiliations:** a Department of Chemistry, University of South Dakota, 414 E. Clark Street, Vermillion, SD 57069, USA

## Abstract

The title compound, C_9_H_7_IN^+^·Cl^−^·2H_2_O, was obtained during the synthesis of 8-iodo­quinoline from 8-amino­quinoline using the Sandmeyer reaction. The 8-iodo­quinolinium ion is almost planar. Solvent water mol­ecules and chloride ions form a hydrogen-bonded chain along the *c* axis *via* O—H⋯Cl links. The 8-iodo­quinolinium ions, which are packed along the *c* axis with cationic aromatic π–π stacking (centroid–centroid distance = 3.624 Å), are linked to the chain *via* N—H⋯O hydrogen bonds.

## Related literature

For the synthesis, see: Lucas & Kennedy (1943 ▶); Sandmeyer (1884 ▶). For a related structure, see: Son & Hoefelmeyer (2008 ▶). For related literature, see: Janiak (2000 ▶).
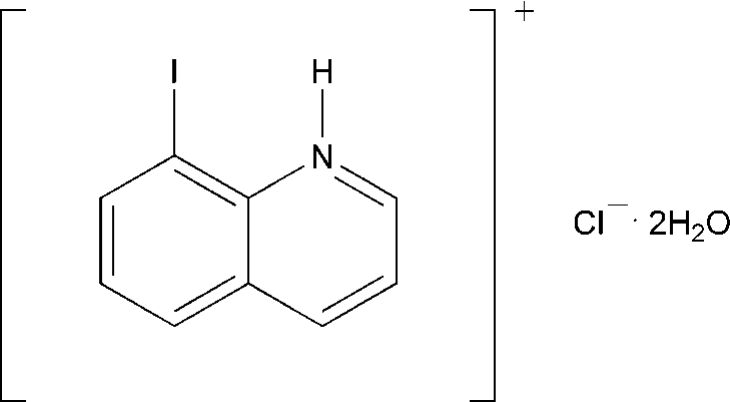


## Experimental

### 

#### Crystal data

C_9_H_7_IN^+^·Cl^−^·2H_2_O
*M* *_r_* = 327.54
Monoclinic, 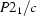

*a* = 8.9600 (18) Å
*b* = 17.580 (4) Å
*c* = 7.1700 (14) Å
β = 97.13 (3)°
*V* = 1120.7 (4) Å^3^
*Z* = 4
Mo *K*α radiation
μ = 3.07 mm^−1^
*T* = 100 (2) K
0.71 × 0.67 × 0.54 mm

#### Data collection

Bruker SMART APEXII diffractometer
Absorption correction: multi-scan (*SADABS*; Bruker, 2006 ▶) *T*_min_ = 0.219, *T*_max_ = 0.288 (expected range = 0.145–0.190)
10655 measured reflections
2045 independent reflections
2040 reflections with *I* > 2σ(*I*)
*R*_int_ = 0.020

#### Refinement

*R*[*F* ^2^ > 2σ(*F* ^2^)] = 0.019
*wR*(*F* ^2^) = 0.046
*S* = 1.25
2045 reflections
143 parameters
6 restraints
H atoms treated by a mixture of independent and constrained refinement
Δρ_max_ = 0.79 e Å^−3^
Δρ_min_ = −0.65 e Å^−3^

### 

Data collection: *APEX2* (Bruker, 2006 ▶); cell refinement: *SAINT* (Bruker, 2006 ▶); data reduction: *SAINT*; program(s) used to solve structure: *SHELXTL* (Sheldrick, 2008 ▶); program(s) used to refine structure: *SHELXTL*; molecular graphics: *ORTEP-3* (Farrugia, 1997 ▶) and *Mercury* (Macrae *et al.*, 2006 ▶); software used to prepare material for publication: *SHELXTL*.

## Supplementary Material

Crystal structure: contains datablocks I, global. DOI: 10.1107/S1600536808031528/ci2652sup1.cif

Structure factors: contains datablocks I. DOI: 10.1107/S1600536808031528/ci2652Isup2.hkl

Additional supplementary materials:  crystallographic information; 3D view; checkCIF report

## Figures and Tables

**Table 1:** Hydrogen-bond geometry (Å, °)

*D*—H⋯*A*	*D*—H	H⋯*A*	*D*⋯*A*	*D*—H⋯*A*
N1—H1⋯O1	0.82 (4)	2.03 (4)	2.755 (3)	147 (3)
N1—H1⋯I1	0.82 (4)	2.85 (4)	3.320 (2)	119 (3)
O1—H1*A*⋯O2	0.84 (1)	1.975 (12)	2.807 (3)	173 (5)
O1—H1*B*⋯Cl1^i^	0.83 (1)	2.75 (3)	3.382 (3)	134 (4)
O2—H2*A*⋯Cl1	0.84 (1)	2.435 (16)	3.237 (2)	160 (3)
O2—H2*B*⋯Cl1^ii^	0.84 (1)	2.379 (12)	3.211 (2)	170 (3)

Symmetry codes: (i) 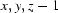; (ii) 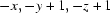.

**Fig. 1.**
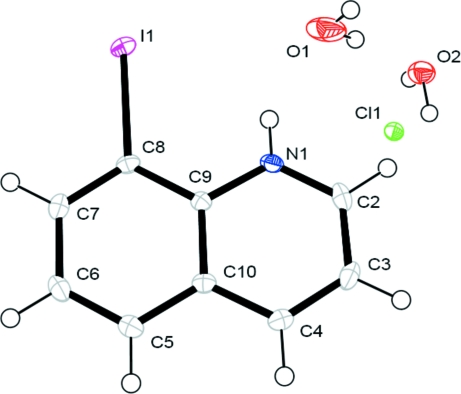
Asymmetric unit of 8-iodoquinolinium chloride dihydrate. Displacement ellipsoids are drawn at the 50% probability level.

**Fig. 2.**
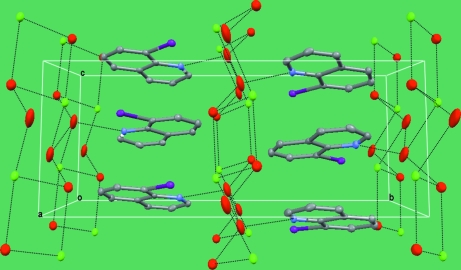
The crystal structure of 8-iodoquinolinium chloride dihydrate, viewed approximately along the a axis. Dotted lines represent hydrogen bonds. H atoms have been omitted for clarity. Displacement ellipsoids are drawn at the 50% probability level.

## supplementary crystallographic information

### Comment

8-Iodoquinoline is a starting material for the synthesis of 8-substituted quinoline derivatives. In this work, 8-iodoquinoline was synthesized starting from 8-aminoquinoline using the Sandmeyer reaction (Sandmeyer, 1884), following the synthesis of iodobenzene (Lucas & Kennedy, 1943). During its synthesis, two 8-iodoquinolinium salt crystals, 8-iodoquinolinium chloride dihydrate and 8-iodoquinolinium triiodide.THF (Son & Hoefelmeyer, 2008) were isolated. The synthesis, characterization and crystal structure of 8-iodoquinolinium chloride dihydrate (Fig. 1) are reported here.

The 8-iodoquinolinium ion is planar, with a maximum deviation of 0.069 (1) Å for the I1 atom. The C8—I1 bond length is 2.110 (3) Å and C9—C8—I1 angle is 121.09 (19) °. A short contact of 3.2083 (10) Å is observed between I1 and Cl1 ion at (1-x, 1-y, 1-z) that is likely due to ion-dipole interaction. The C8—I1···Cl1 angle is almost linear (177.13 (8)°).

Lattice water molecules and chloride ions form an extended hydrogen bonding chain network along the *c* axis (Table 1). Hydrogen bonding four-membered rings comprising O2 and Cl1 are alternately sharing edges with six-membered rings (in chair form) comprising O2, C1l and O1 along the *c* axis (Fig. 2). Atom O1 of the six-membered ring is hydrogen-bonded to atom N1 of the quinolinium ion. The 8-iodoquinolinium ions are parallel to each other and form a π-stack that is propogated along the *c* axis. The π-π stacking distance between the 8-iodoquinolinium rings is 3.624 Å (centroid-centroid distance between the 8-iodoquinolinium rings); there may be weak cationic repulsion between the rings (Janiak, 2000).

### Experimental

A mixture of 8-aminoquinoline (10 g, 0.069 mol) and water (50 ml) was heated with stirring. The mixture was cooled in an ice bath and concentrated HCl (50 ml) was added to form a red solution. An ice-cooled NaNO_2_ (7.8 g, 0.113 mol) solution in water (50 ml) was slowly transferred to the 8-aminoquinoline solution. A light brown precipitate was formed during the addition step but eventually it disappeared to form a reddish transparent solution. KI (17.9 g, 0.108 mol) dissolved in water (25 ml) was then added to the reaction mixture. Bubbles and brownish vapour evolved during the addition. The solution turned to dark brown with a black precipitate. The solution was then refluxed with a watch glass on top of the beaker, and it turned reddish brown with formation of a heavy organic layer; the black precipitate remained. After cooling and standing overnight, golden brown crystals of 8-iodoquinolinium chloride dihydrate had formed spontaneously in the solution. The mixture was neutralized upon addition of NaOH solution, which led to dissolution of the golden brown crystals and retention of the black precipitate. The liquid portion was separated from the black precipitate. 8-iodoquinoline was recoverd from the liquid portion upon extraction with toluene. Yield: 10.71 g of 8-iodoquinoline (61%). Physical data for 8-iodoquinolinium chloride dihydrate: m.p. 388-390 K (431-433 K after dehydration). ^1^H NMR (methanol-*d4*): 7.638–7.716 (dd, 1H, quin C*H*), 8.144–8.214 (dd, 1H, quin C*H*), 8.313–8.360 (dd, 1H, quin C*H*), 8.605–8.647 (dd, 1H, quin C*H*), 9.179–9.228 (dd, 1H, quin C*H*), 9.253–9.288 (dd, 1H, quin C*H*). ^13^C NMR (methanol-*d4*): 90.253 (quin *C*8), 124.038 (quin *C*H), 131.224(quin *C*H), 131.475 (quin *C*9/10), 132.070 (quin *C*H), 139.889 (quin *C*9/10), 147.113(quin *C*H), 148.440 (quin *C*H), 149.608 (quin *C*H). Analysis calculated for C_9_H_7_ClIN (dehydrated): C 37.08, H 2.42, N 4.80%; found: C 36.76, H 2.40, N 4.85%.

### Refinement

The water H atoms were located in a difference map and refined with O-H and H···H distance restraints of 0.84 (1) Å and 1.37 (2) Å, respectively, and with *U*_iso_(H) = 1.5*U*_eq_(O). The N-bound H atom was also located in a difference map and refind freely. C-bound H atoms were positioned geometrically (C-H = 0.93 Å) and allowed to ride on the parent atoms with *U*_iso_(H) = 1.2*U*_eq_(C).

### Crystal data

**Table:**

C_9_H_7_IN^+^·Cl^−^·2H_2_O	*F*(000) = 632
*M**_r_* = 327.54	*D*_x_ = 1.941 Mg m^−^^3^
Monoclinic, *P*2_1_/*c*	Melting point: 388 K
Hall symbol: -P 2ybc	Mo *K*α radiation, λ = 0.71073 Å
*a* = 8.9600 (18) Å	Cell parameters from 9991 reflections
*b* = 17.580 (4) Å	θ = 2.3–28.6°
*c* = 7.1700 (14) Å	µ = 3.07 mm^−^^1^
β = 97.13 (3)°	*T* = 100 K
*V* = 1120.7 (4) Å^3^	Block, brown
*Z* = 4	0.71 × 0.67 × 0.54 mm

### Data collection

**Table:**

Bruker SMART APEXII diffractometer	2045 independent reflections
Radiation source: fine-focus sealed tube	2040 reflections with *I* > 2σ(*I*)
graphite	*R*_int_ = 0.020
ω scans	θ_max_ = 25.4°, θ_min_ = 2.3°
Absorption correction: multi-scan (SADABS; Bruker, 2003)	*h* = −10→10
*T*_min_ = 0.219, *T*_max_ = 0.288	*k* = −21→21
10655 measured reflections	*l* = −8→8

### Refinement

**Table:**

Refinement on *F*^2^	Primary atom site location: structure-invariant direct methods
Least-squares matrix: full	Secondary atom site location: difference Fourier map
*R*[*F*^2^ > 2σ(*F*^2^)] = 0.019	Hydrogen site location: inferred from neighbouring sites
*wR*(*F*^2^) = 0.046	H atoms treated by a mixture of independent and constrained refinement
*S* = 1.25	*w* = 1/[σ^2^(*F*_o_^2^) + (0.0437*P*)^2^ + 2.836*P*] where *P* = (*F*_o_^2^ + 2*F*_c_^2^)/3
2045 reflections	(Δ/σ)_max_ = 0.001
143 parameters	Δρ_max_ = 0.79 e Å^−^^3^
6 restraints	Δρ_min_ = −0.65 e Å^−^^3^

### Special details

**Table:**

Geometry. All e.s.d.'s (except the e.s.d. in the dihedral angle between two l.s. planes) are estimated using the full covariance matrix. The cell e.s.d.'s are taken into account individually in the estimation of e.s.d.'s in distances, angles and torsion angles; correlations between e.s.d.'s in cell parameters are only used when they are defined by crystal symmetry. An approximate (isotropic) treatment of cell e.s.d.'s is used for estimating e.s.d.'s involving l.s. planes.
Refinement. Refinement of *F*^2^ against ALL reflections. The weighted *R*-factor *wR* and goodness of fit *S* are based on *F*^2^, conventional *R*-factors *R* are based on *F*, with *F* set to zero for negative *F*^2^. The threshold expression of *F*^2^ > σ(*F*^2^) is used only for calculating *R*-factors(gt) *etc*. and is not relevant to the choice of reflections for refinement. *R*-factors based on *F*^2^ are statistically about twice as large as those based on *F*, and *R*- factors based on ALL data will be even larger.

### Fractional atomic coordinates and isotropic or equivalent isotropic displacement parameters (Å^2^)

**Table:**

	*x*	*y*	*z*	*U*_iso_*/*U*_eq_	
C2	0.1207 (3)	0.35738 (15)	−0.0118 (4)	0.0175 (6)	
H2	0.0968	0.4100	−0.0215	0.021*	
C3	0.0078 (3)	0.30408 (17)	−0.0595 (4)	0.0187 (6)	
H3	−0.0918	0.3199	−0.1035	0.022*	
C4	0.0429 (3)	0.22795 (15)	−0.0418 (4)	0.0155 (5)	
H4	−0.0333	0.1909	−0.0724	0.019*	
C5	0.2333 (3)	0.12642 (15)	0.0433 (4)	0.0170 (5)	
H5	0.1591	0.0881	0.0155	0.020*	
C6	0.3795 (3)	0.10627 (15)	0.1040 (4)	0.0190 (6)	
H6	0.4053	0.0541	0.1199	0.023*	
C7	0.4923 (3)	0.16273 (16)	0.1430 (4)	0.0169 (5)	
H7	0.5932	0.1477	0.1821	0.020*	
C8	0.4574 (3)	0.23944 (15)	0.1248 (4)	0.0134 (5)	
C9	0.3060 (3)	0.26110 (14)	0.0660 (3)	0.0115 (5)	
C10	0.1928 (3)	0.20430 (15)	0.0220 (4)	0.0132 (5)	
N1	0.2614 (3)	0.33639 (13)	0.0471 (3)	0.0143 (5)	
H1	0.326 (4)	0.369 (2)	0.080 (5)	0.029 (10)*	
Cl1	0.12024 (7)	0.54723 (4)	0.76556 (9)	0.01743 (14)	
I1	0.629687 (18)	0.321563 (10)	0.17166 (2)	0.01562 (7)	
O1	0.3734 (3)	0.48091 (13)	0.1171 (4)	0.0404 (6)	
H1A	0.320 (4)	0.506 (2)	0.183 (5)	0.061*	
H1B	0.363 (5)	0.498 (2)	0.008 (3)	0.061*	
O2	0.1749 (2)	0.55620 (12)	0.3282 (3)	0.0240 (4)	
H2A	0.183 (4)	0.559 (2)	0.4461 (15)	0.036*	
H2B	0.104 (3)	0.5262 (17)	0.294 (4)	0.036*	

### Atomic displacement parameters (Å^2^)

**Table:**

	*U*^11^	*U*^22^	*U*^33^	*U*^12^	*U*^13^	*U*^23^
C2	0.0231 (14)	0.0138 (13)	0.0161 (13)	0.0066 (11)	0.0044 (11)	0.0019 (10)
C3	0.0122 (13)	0.0270 (15)	0.0173 (14)	0.0054 (11)	0.0034 (11)	0.0065 (11)
C4	0.0139 (13)	0.0186 (13)	0.0138 (12)	−0.0033 (10)	0.0014 (10)	−0.0002 (10)
C5	0.0197 (14)	0.0140 (13)	0.0177 (13)	−0.0021 (11)	0.0034 (11)	−0.0005 (10)
C6	0.0231 (15)	0.0126 (13)	0.0217 (14)	0.0031 (11)	0.0041 (11)	0.0006 (11)
C7	0.0141 (13)	0.0192 (13)	0.0174 (13)	0.0039 (11)	0.0024 (10)	0.0004 (11)
C8	0.0121 (12)	0.0158 (13)	0.0124 (12)	−0.0034 (10)	0.0016 (10)	−0.0024 (10)
C9	0.0130 (12)	0.0124 (12)	0.0099 (11)	−0.0005 (10)	0.0041 (9)	0.0003 (9)
C10	0.0143 (13)	0.0149 (12)	0.0111 (12)	−0.0022 (10)	0.0041 (10)	−0.0005 (10)
N1	0.0153 (12)	0.0110 (11)	0.0172 (12)	−0.0028 (9)	0.0043 (9)	0.0004 (9)
Cl1	0.0163 (3)	0.0132 (3)	0.0221 (3)	−0.0013 (2)	0.0002 (2)	−0.0013 (2)
I1	0.01147 (10)	0.01954 (11)	0.01569 (11)	−0.00378 (6)	0.00106 (7)	−0.00135 (6)
O1	0.0416 (15)	0.0188 (12)	0.0660 (18)	−0.0115 (10)	0.0268 (14)	−0.0137 (11)
O2	0.0235 (11)	0.0214 (11)	0.0267 (11)	−0.0048 (8)	0.0017 (9)	−0.0003 (9)

### Geometric parameters (Å, °)

**Table:**

C2—N1	1.331 (4)	C7—C8	1.387 (4)
C2—C3	1.390 (4)	C7—H7	0.95
C2—H2	0.95	C8—C9	1.421 (4)
C3—C4	1.377 (4)	C8—I1	2.110 (3)
C3—H3	0.95	C9—N1	1.384 (3)
C4—C10	1.426 (4)	C9—C10	1.430 (4)
C4—H4	0.95	N1—H1	0.82 (4)
C5—C6	1.374 (4)	O1—H1A	0.836 (10)
C5—C10	1.420 (4)	O1—H1B	0.834 (10)
C5—H5	0.95	O2—H2A	0.840 (10)
C6—C7	1.419 (4)	O2—H2B	0.841 (10)
C6—H6	0.95		
			
N1—C2—C3	121.5 (2)	C8—C7—H7	119.5
N1—C2—H2	119.2	C6—C7—H7	119.5
C3—C2—H2	119.2	C7—C8—C9	119.0 (2)
C4—C3—C2	118.8 (2)	C7—C8—I1	119.89 (19)
C4—C3—H3	120.6	C9—C8—I1	121.09 (19)
C2—C3—H3	120.6	N1—C9—C8	122.6 (2)
C3—C4—C10	120.6 (2)	N1—C9—C10	117.2 (2)
C3—C4—H4	119.7	C8—C9—C10	120.2 (2)
C10—C4—H4	119.7	C5—C10—C4	122.3 (2)
C6—C5—C10	120.2 (2)	C5—C10—C9	119.0 (2)
C6—C5—H5	119.9	C4—C10—C9	118.8 (2)
C10—C5—H5	119.9	C2—N1—C9	123.1 (2)
C5—C6—C7	120.6 (3)	C2—N1—H1	120 (3)
C5—C6—H6	119.7	C9—N1—H1	117 (3)
C7—C6—H6	119.7	H1A—O1—H1B	110 (2)
C8—C7—C6	121.0 (2)	H2A—O2—H2B	107 (2)
			
N1—C2—C3—C4	1.1 (4)	C6—C5—C10—C9	−0.5 (4)
C2—C3—C4—C10	−0.8 (4)	C3—C4—C10—C5	179.6 (3)
C10—C5—C6—C7	−1.1 (4)	C3—C4—C10—C9	−0.4 (4)
C5—C6—C7—C8	1.4 (4)	N1—C9—C10—C5	−178.7 (2)
C6—C7—C8—C9	0.0 (4)	C8—C9—C10—C5	1.8 (4)
C6—C7—C8—I1	−177.1 (2)	N1—C9—C10—C4	1.3 (3)
C7—C8—C9—N1	179.0 (2)	C8—C9—C10—C4	−178.2 (2)
I1—C8—C9—N1	−3.9 (3)	C3—C2—N1—C9	−0.2 (4)
C7—C8—C9—C10	−1.6 (4)	C8—C9—N1—C2	178.4 (2)
I1—C8—C9—C10	175.50 (18)	C10—C9—N1—C2	−1.0 (4)
C6—C5—C10—C4	179.5 (2)		

### Hydrogen-bond geometry (Å, °)

**Table:**

*D*—H···*A*	*D*—H	H···*A*	*D*···*A*	*D*—H···*A*
N1—H1···O1	0.82 (4)	2.03 (4)	2.755 (3)	147 (3)
N1—H1···I1	0.82 (4)	2.85 (4)	3.320 (2)	119 (3)
O1—H1A···O2	0.84 (1)	1.98 (1)	2.807 (3)	173 (5)
O1—H1B···Cl1^i^	0.83 (1)	2.75 (3)	3.382 (3)	134 (4)
O2—H2A···Cl1	0.84 (1)	2.44 (2)	3.237 (2)	160 (3)
O2—H2B···Cl1^ii^	0.84 (1)	2.38 (1)	3.211 (2)	170 (3)

Symmetry codes: (i) *x*, *y*, *z*−1; (ii) −*x*, −*y*+1, −*z*+1.
